# Is Pediatric Dentistry a Topic of Interest for Pediatric Journals? A Scoping Review

**DOI:** 10.3390/children8090720

**Published:** 2021-08-24

**Authors:** Maria Grazia Cagetti, Araxi Balian, Silvia Cirio, Nicole Camoni, Claudia Salerno, Gianluca Martino Tartaglia

**Affiliations:** 1 Department of Biomedical, Surgical and Dental Sciences, University of Milan, 20122 Milan, Italy; maria.cagetti@unimi.it (M.G.C.); silviacirio@alice.it (S.C.); n.camoni@gmail.com (N.C.); claudia.salerno@ymail.com (C.S.); gianluca.tartaglia@unimi.it (G.M.T.); 2 UOC Maxillo-Facial Surgery and Dentistry Fondazione IRCCS Cà Granda, Ospedale Maggiore Policlinico, University of Milan, 20100 Milan, Italy

**Keywords:** pediatric dentistry, pediatrics, bibliometric analysis

## Abstract

Background: Pediatric dentistry shares many skills with pediatrics. This review evaluates the amount of literature on pediatric dentistry in the first 30 pediatric journals classified by the Web of Science in 2019. The aim was to perform a quantitative analysis of the main dental topics addressed. Methods: A scoping review with the PRISMA-ScR criteria was performed. The Clarivate Analytics Journal Citation Report was consulted for journals ranked in the category “Pediatrics” in 2019. Papers were searched in PubMed using an ad hoc prepared string. Results: A total of 504 papers were included. Papers on dental hard tissues were the most prevalent (45.6%), followed by dental public health (23.2%), orofacial development (15.3%), soft tissues related conditions (12.3%), and orofacial trauma (3.6%). Increasing trends have been observed for total papers published (R^2^ = 0.9822) and total dental papers (R^2^ = 0.8862), with no statistically significant differences (χ^2^(6) = 0.051 *p* > 0.05). The majority of papers (*n* = 292, 57.9%) were cited between 1 and 10 times, whilst less than 7% of papers received more than 40 citations. Discussion: It is desirable that papers on pediatric dentistry increase in the pediatric scenario, allowing the two related disciplines to intertwine more in the future.

## 1. Introduction

“Pediatrics”, according to PubMed, is a medical specialty concerned with maintaining health and providing medical care to children from birth to adolescence. Oral health is an essential part of overall health and wellbeing. Good oral health needs not only sound teeth and periodontium, but also the wellbeing of all structures of the mouth, jaw, oral, and peri-oral tissues. Most oral health conditions are largely preventable in children and can be treated in their early stages. Caries represent one of the most common non-communicable diseases [1] and the most prevalent among oral diseases in childhood, despite being easily prevented [2,3]. Factors contributing to oral diseases are an unhealthy diet (rich in fermentable sugars), inadequate oral hygiene, and low fluoride exposure [4,5,6].

The prevalence of untreated dental caries is high worldwide. Data from the Global Burden of Disease (GBD) 2015 show that 3.5 billion people live with dental health-related issues globally [1,7]. Nevertheless, essential oral care is still not provided to many children, especially in low and middle-income countries [8,9,10,11]. According to WHO, treatment for oral health conditions is expensive and usually not part of universal health coverage; in most high-income countries, dental treatment averages 5% of total health expenditure and 20% of out-of-pocket health expenditure [4]. In addition to caries, it is very common that hard dental tissues have developmental defects of enamel (DDE) and dentine, which can affect both primary and permanent dentitions [12]. The ameloblasts, the cells that produce enamel, are very sensitive to any local/systemic alteration, even if it lasts a few days [13,14]. Common DDE are qualitative enamel alterations like Molar Incisor Hypomineralization (MIH), affecting 13–14% of children worldwide [15,16]. Dental traumas are also a frequent childhood experience; the prevalence occurs from 6.1% to 62.1% in toddlers and pre-school children [17], and from 5.3% to 21.0% in schoolchildren [18]. Regarding periodontium diseases, plaque-induced gingivitis has been reported in the majority of samples of adolescents screened worldwide, with a prevalence of about 30%. Gingival inflammation is a well-documented disease in children and adolescents, especially with poor oral hygiene and those affected by systemic pathologies like diabetes or leukemia [19,20,21]. Untreated gingivitis, later in life, is highly connected to periodontitis development and tooth loss [22,23]. Teeth and jaws are the keys of the occlusion, and malocclusion conditions are well spread in all ethnic groups [24], with between 57% and 59% of the population need some degree of orthodontic treatment [25]. Many studies have shown an association between malocclusion and worse oral health-related quality of life in children and adolescents [26], due to the functional and social importance of the oral cavity. It appears evident that reaching adequate oral health is a global challenge that can’t be achieved by dental professionals alone [8,27,28].

Dentists are the specialists of oral health. In the majority of countries, they are enrolled in a separate faculty from medical students. During the past decades, dentistry developed in many hyper specialties, and pediatric dentistry is one of them. Anyway, this evolution leads to an estrangement of highly related branches of medicine like pediatric dentistry and pediatrics [28,29]. These specializations should share common knowledge to reach comprehensive treatments for children, but this is often unreal, due to their impressive detachment. This separation, which starts from university, develops in different paths of post-degree courses and updates. Nowadays, most practitioners commonly use online databases, such as PubMed and official flagship journals of the main scientific organizations, to find information that would increase their competencies in everyday practice [30]. Insufficient knowledge of pediatric personnel (pediatrics, midwives, and pediatric nurses) on the preventive and mini-invasive dental/oral treatments have been reported to lead to a late referral to the pediatric dentist. An overall higher biological and economic cost for public health institutions and families was also reported [31]. This could also be the consequence of a lack of sufficient production of papers on dental topics in dedicated journals, but no evidence is available so far on this issue.

The scoping review evaluates the amount of literature published on pediatric dentistry in the first 30 pediatric journals classified by the Web of Science (WOS) in 2019. In addition, a quantitative analysis of the main dental topics addressed over time will be presented.

## 2. Materials and Methods

### 2.1. Protocol

A scoping review was designed and performed to investigate the literature addressing dental topics published in pediatric journals. The review followed the Preferred Reporting Items for Systematic reviews and Meta-Analyses extension for Scoping Reviews (PRISMA-ScR) guidelines (Table S1 PRISMA-ScR Checklist) [32].

### 2.2. Eligibility Criteria

The inclusion criteria were:

- Type of study: Review/meta-analysis, guidelines/consensus paper, interventional studies, observational studies, case report/case series;
- Publication languages: No languages restriction applied;
- Time of publication: No time restriction applied;
- Topics: Any papers regarding pediatric dentistry issues as the primary or not primary outcome, including diseases of hard and soft oral tissues, orofacial trauma, orofacial development, dental public health;
- Outcomes considered: Number of papers, types of studies, topic addressed, number of citations.

### 2.3. Information Sources and Search Strategy

The Clarivate Analytics Journal Citation Report (JCR) was consulted in April 2021 (S.C.) to retrieve journals ranked in the category “Pediatrics” in 2019, which was the last year JCR had been released at that time. The top 30 journals with the highest impact factor score and indexed in Medline were selected. 

Papers were searched in Medline electronic database via PubMed from the inception until 31 May 2021, using the following search string:

(“dental”[Title/Abstract] OR “dental caries”[Title/Abstract] OR “enamel defects”[Title/Abstract] OR “oral mucosal pathology”[Title/Abstract] OR “oral mucosal lesions”[Title/Abstract] OR “gingivitis”[Title/Abstract] OR “stomatitis”[Title/Abstract] OR “orthodontic “[Title/Abstract] OR “jaw development”[Title/Abstract] OR “jaw growth”[Title/Abstract] OR “oral anomalies”[Title/Abstract] OR “facial anomalies”[Title/Abstract] OR “malocclusion “[Title/Abstract] OR “dental trauma”[Title/Abstract] OR “oral trauma”[Title/Abstract] OR “oral injuries”[Title/Abstract] OR “maxillary trauma”[Title/Abstract] OR “jaw trauma”[Title/Abstract]) AND (“*journal title*”[Journal]). In case a journal has changed its name over time, the search string with the previous journal titles has been also searched (Table 1). 

### 2.4. Study Selection

The output of the references searched was uploaded into Excel^TM^ software 16.16 (Microsoft, Redmond, DC, USA). Four authors (A.B., S.C., C.S., and N.C.) independently examined all the abstracts to establish whether each paper should or should not be included in the scoping review. Disagreements were resolved through discussion in doubtful cases. Where resolution was not possible, another author was consulted (M.G.C.).

### 2.5. Charting the Data 

A structured template for charting the data was designed (G.M.T.). The data extraction form was realized without masking the name of the journal, title, and authors.

The data extraction form contains and summarizes the main characteristics of each paper: journal title, journal impact factor, total journal citations, paper title, authors, publication year, PubMed Uniform Resource Locator, country, area, topic, type of study, oral health primary outcome, number of citations.

Dental topics were grouped into five areas as follows:

- Hard tissues: caries prevention, caries treatment, caries epidemiology, developmental defects of enamel (DDE) and dentine;
- Soft tissues: oral aphthae, oral infections, gingivitis, and other soft tissues disease;
- Orofacial trauma: dental trauma, jaws trauma, combined trauma (dental and/or jaws and/or soft tissues), suspected abuse;
- Orofacial development: orofacial anomalies in syndromic and not-syndromic patients;
- Dental Public Health: health insurance, health services, pediatrician’s role.

Moreover, papers were divided into two categories, those with oral health as the primary outcome, and those including an oral health issue within other outcomes.

Finally, an author (C.S.) checked every extraction for accuracy and completeness.

### 2.6. Collating, Summarizing, and Analyzing Data

Data extracted from the studies were exported to an Excel spreadsheet (Microsoft Office 365^®^, Redmond, WA, USA). Included studies were not subjected to critical appraisal, since the objective of the review was to evaluate the amount of literature published on pediatric dentistry in pediatric journals.

Descriptive analysis was performed using the same software, and trends estimations of the number of papers across time were calculated. A chi-square test was performed for multiple comparisons between the total number of papers and those regarding dental topics over time.

Caries prevalence data from the Global Health Data Exchange tool [33] were recorded and compared to the number of papers published on caries issues.

## 3. Results

**Figure 1:**
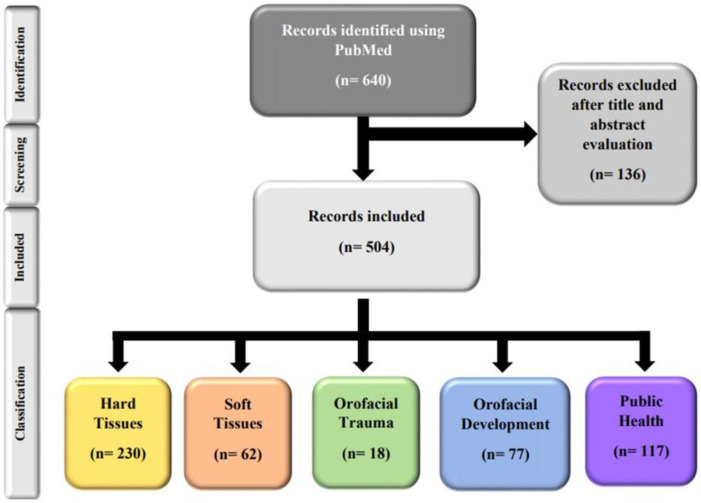
PRISMA flow diagram.

A total of 640 papers were identified by PubMed search. Then, 136 were excluded following the title and abstract screening (Table S2), leaving 504 papers for consideration (Table S3). Figure 1 shows the PRISMA flow diagram of the selection process and the classification process by area of the included papers.

### 3.1. Journals: Features and Relevance

Twelve of the top 30 journals ranked in JCR in 2019 had changed their name over time: Fifty journal titles were identified from 1940 to date by the National Library of Medicine catalog, 35 titles of which were indexed in Medline (Table 1). More than 70% of all included articles were retrieved from only five pediatric journals—25% (*n* = 133) were published in “Pediatrics”, which was also the journal that published over time the highest numbers of papers (*n* = 37,582), 17% (*n* = 87) in “The Journal of Pediatrics”, and 31% (*n* = 156) in other three journals (“JAMA Pediatrics”, “Archives of Diseases in Childhood”, and “Academic Pediatrics”). Among the other journals, from twenty of them, less than 25 papers each were retrieved, and from the last five, no paper was found. Journals addressing general pediatric medicine had higher numbers of papers on dental topics than those publishing papers related to a specific field within pediatrics (Table 1).

### 3.2. Most Frequent Areas and Topics

Table 2 summarizes the included papers per area and per topic. Among the selected papers, those belonging to the dental hard tissues area were the most prevalent (45.6%), followed by those in the dental public health area (23.2%), the orofacial development area (15.3%), the soft tissues related conditions area (12.3%) and finally the orofacial trauma area (3.6%). Regarding the most recurrent topics for each area, caries prevention (37.0%) was the most prevalent in the hard tissues area, syndromic children (72.7%) in that on orofacial development, oral aphthae (51.6%) in soft tissues, health services (54.7%) in public health, and finally, suspected abuse (38.9%) was the most prevalent topic in the orofacial trauma area. The majority of the included papers had the dental topic as the primary outcome (71.4%).

**Figure 2:**
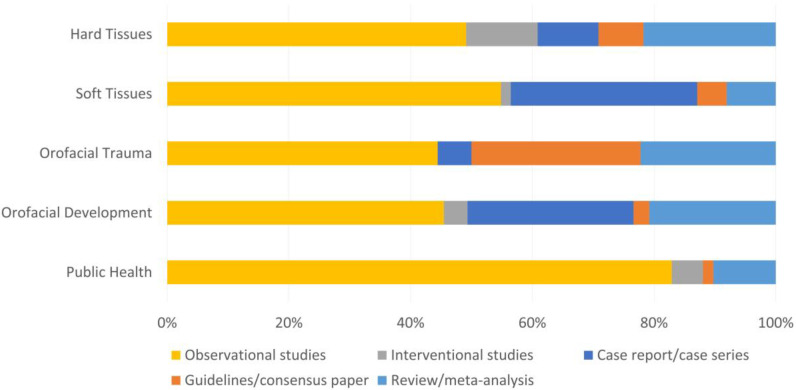
Type of studies of the included papers in different dental areas.

An observational study was the most prevalent type of study in all areas, ranging from 44.5% in the orofacial trauma area to 82.9% in the public health area (Figure 2). Guidelines papers reached the highest value in orofacial trauma (27.7%), whilst case reports were more prevalent in soft tissues area (30.7%) and orofacial development area (27.3%).

**Figure 3:**
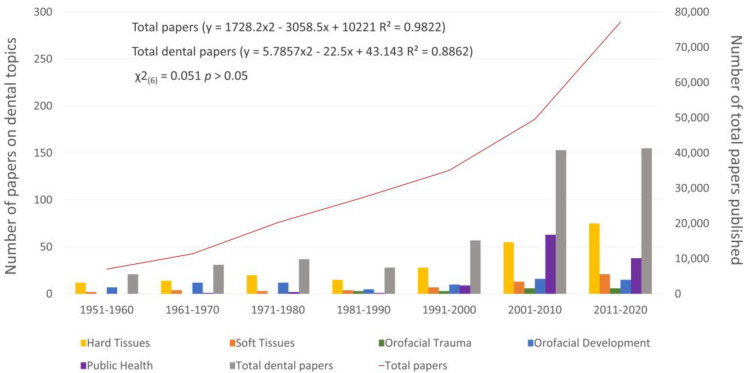
Number of total papers, number of papers on dental issues (total and per area) published in pediatric journals over the last seven decades (1951–2020). Trend coefficients are reported for the total papers and total dental papers.

Figure 3 displays the publication trends of papers on dental topics and total papers published in the selected pediatrics journals over the last seven decades (1951–2020). An increasing trend has been observed for both variables (R^2^ = 0.9822 for total papers and R^2^ = 0.8862 for total dental papers) with no significant differences (χ^2^(6) = 0.051 *p* > 0.05), although the rise in numbers of papers on dental issues has been delayed, starting from the 1990s. Within papers on dental issues, those featuring hard tissues and public health issues mostly contributed to the publication trend in the last three decades (Figure 3).

### 3.3. Caries Papers and Caries Global Burden

**Figure 4:**
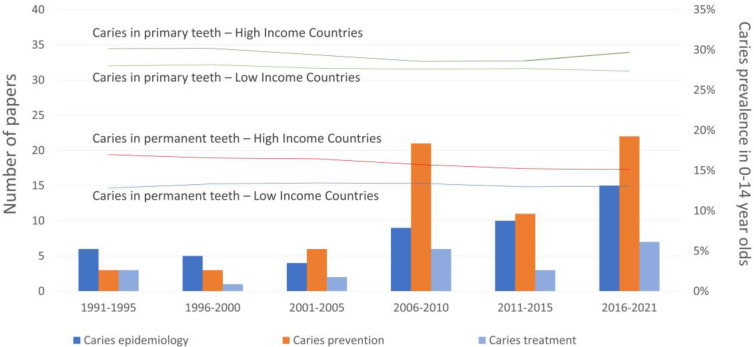
Publication trend on caries topics and caries prevalence in children from high- and low-income countries (data from the Global Health Data Exchange website, 1991–2021).

Since caries topics count for 36.3% of the total amount of papers on pediatric dentistry issues, the tendency of caries topics through time and the epidemiological trend of the disease in childhood (0–14 years old children) for the two dentitions was investigated. Caries data were obtained from the Global Burden Diseases Database available from 1991 (Figure 4). Caries prevalence lowered in 2005 and increased in 2015, particularly in primary teeth in high-income countries, according to the number of papers published on caries prevention and treatment. Papers on caries epidemiology continued to increase over time.

### 3.4. Citation Results

**Figure 5:**
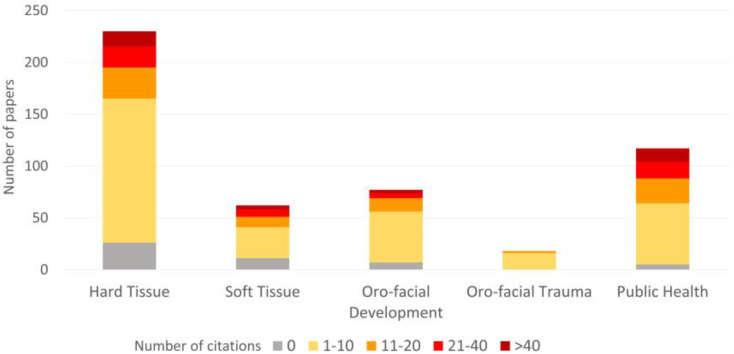
Number of citations of the included papers by area.

The majority of papers (*n* = 292, 57.9%) were cited between 1 and 10 times, whilst less than 7% of papers received more than 40 citations. A small group (*n* = 46, 9.1%) of papers have no citations, and only four papers have obtained more than 100 citations (Table S3). The citations distribution of the included studies is displayed by area and topics in Table 3 and Figure 5. Papers on hard tissues and public health received the highest number of citations (*n* = 2762, 43.9% and *n* = 2028, 32.3%, respectively), with caries prevention and health insurance as the most cited topics (17.8% and 14.1% of the total citations, respectively). Papers focusing on oral health as the primary outcome were cited more than those in which it was not, except for soft tissues and orofacial development areas. The highest average number of citations per paper was observed for public health area (mean = 17.3), followed by hard tissues (mean = 12.0), soft tissues (mean = 11.9), orofacial development (mean= 8.8), and orofacial trauma (mean = 4.6) area. All areas, except for orofacial trauma, included papers with a number of citations greater than 40; nevertheless, orofacial trauma did not include any paper with no citation.

## 4. Discussion 

This scoping review highlights the shortage of papers on pediatric dentistry issues in pediatric journals. In fact, although papers on dental topics increased over time, especially in the last two decades, the number of papers remains small if compared to the impressive increase of the overall number of papers in the pediatric journals considered.

A considerable asymmetry in the distribution of papers on dental issues among the thirty journals considered was observed. The majority of papers had been published by only five general pediatric journals, while those dedicated to specific areas seem less interested in publishing oral health issues. Surprisingly, only one paper was found in journals dedicated to childhood obesity, even if multiple aspects and shared risk factors link oral pathologies and the systemic condition [34,35]. This could be explained because pediatric dentistry is believed as a third-party competence. Pediatricians should be better informed about the close interconnection between systemic diseases and their influence on oral health. Similarly, dentists should not only focus on the diagnosis and therapy of dental disease, but contextualize it in the overall clinical picture [36,37]. 

Observational studies were the most prevalent type of study in all dental areas considered. Observational studies offer advantages as the relatively quick and low-cost carrying-out, and can be used to study multiple outcomes simultaneously. However, the main limit is that they cannot differentiate between cause and effect or within the sequence of events, and so they are not considered high-quality design studies. Nevertheless, because this study aims to measure risk factors or to collect exposure data related to a disease, as for many papers on dental topics, observational studies are often suitable [38].

The most common topic of papers published in pediatric journals was caries prevention; on that note, the number of papers seems to follow caries prevalence variation over time. 

Although a high number of papers published on caries prevention in the last decade were found, caries data still highlights a high prevalence of lesions in primary teeth. This situation requires a great collective political, economic and medical effort, and the knowledge deriving from scientific literature can only represent the starting point. Moreover, an effort should also be advocated in improving communication and cooperation among practitioners who care for children to share multi-specialist knowledge [30].

The majority of the papers on dental topics amounted to less than ten citations each. This data is in line with the number of citations found for the top 100 most-cited papers published in pediatric dentistry journals in 2019, where only seven papers were cited more than 100 times with a range between 42 and 182 citations [39]. An interesting data, might be that the highest number of citations in proportion to the number of papers included, was found in public health areas, particularly regarding health insurance and health services topics. These topics have probably become of greater scientific interest for pediatric personnel with the introduction in the US of state insurance programs, aimed at children belonging to low-income families. The Children’s Health Insurance Program (CHIP) was established in 1997 and provides health coverage to children in families with incomes too high to qualify for Medicaid, but who can’t afford private insurances. The CHIP/Medicaid provides health coverage, including dental cares, to children and young people less than 21 years of age [40].

Some limitations of this review need to be considered—firstly, the choice of the journals included. Papers published in the first top 30 out of 128 total pediatrics journals ranked in 2019 JCR were included. This choice could have excluded eligible papers and might have influenced the final results. Journals searched represent nearly 25% of all pediatric journals ranked in 2019 and were all indexed in the 2018 and 2020 JCR. Secondly, the use of a bibliometric ranking to select the journals avoids the introduction of selection bias. Within this limit, it can be assumed that the journals and papers selected can be considered representative for the category, and results provide a reliable, though restricted, overview of dental publications in pediatric journals that fits the purpose of this scoping review. Thirdly, this review also included papers in which the dental topic was not the primary outcome, but only one of the aspects considered. This choice led to an enlargement of the included sample by about a quarter. On the one hand, if this inclusion may seem a selection bias, on the other, it allows the inclusion of multidisciplinary papers that are extremely useful for a correct integrated management of young patients. 

A strength of this review was that it was the first, at best of authors’ knowledge, aiming to verify how much two related disciplines share scientific knowledge. This aspect is becoming more important day after day, due to knowledge advancement’s speed in medical fields, requiring constant updates not only on specific issues of each medical branch, but also on related issues. The scientific literature offers a unique chance to obtain constantly update information in an easy and economical way.

In conclusion, this scoping review provides an overview of dental publications in the first 30 pediatric journals according to the WOS impact factor. The results show a small number of dental publications compared to the total amount of published papers. The areas of greatest pediatric interest in the dental field seem to be caries and dental public health-related issues. The papers in these two areas were even the most cited. It is desirable that papers on pediatric dentistry issues arise in number and interest in the pediatric scientific scenario, allowing the two related disciplines to intertwine more in the future.

## Figures and Tables

**Table 1:** Bibliometric characteristics of the included pediatric journals and PubMed research results at May 2021.

WOS Rank 2019				PubMed Results at May 2021		
	Journal Title and Previous Publication’s Names (Publication Period)	Journal IF	Total Citation	Total Papers Published	Included Papers	Excluded Papers
			*n*	*n*	*n*	*n*
1	JAMA Pediatrics (2013–) Archives of pediatrics and adolescent medicine (1994–2012) American journal of diseases of children (1960–1993) A.M.A. journal of diseases of children (1956–1960) A.M.A. American journal of diseases of children (1950–1955) American journal of diseases of children (1911–1950)	13.946	9795	19,216	47	12
2	Lancet Child and Adolescent Health (2017–)	8.543	979	830	0	2
3	Journal of the American Academy of Child and Adolescent Psychiatry (1987–) Journal of the American Academy of Child Psychiatry (1962–1986)	6.936	19,831	8657	0	1
4	Archives of Disease in Childhood-Fetal and Neonatal Edition (1988–)	5.436	4868	4948	3	0
5	Pediatrics (1948–)	5.359	79,434	37,582	133	67
6	Pediatric Allergy and Immunology (1990–)	4.699	4456	3560	3	1
7	Developmental Medicine and Child Neurology (1962–) Cerebral palsy bulletin (1958–1961)	4.406	13,007	11,004	18	2
8	Journal of Adolescent Health (1991–) Journal of adolescent health care (1980–1990)	3.945	16,287	7852	21	5
9	European Child and Adolescent Psychiatry (1992–)	3.941	5422	3506	1	0
10	The Journal of Pediatrics (1932–) Transactions (1931–1931)	3.700	31,902	35,780	87	20
11	Seminars in Fetal and Neonatal Medicine (2004–) Seminars in neonatology (1996–2004)	3.540	2583	1548	1	1
12	Clinics in Perinatology (1974–)	3.519	2557	2525	2	0
13	Pediatric Obesity (2012–) International journal of pediatric obesity (2006–2011)	3.429	2306	1869	1	0
14	Seminars in Perinatology (1977–)	3.231	3400	2554	0	2
15	Pediatrics Diabetes (2000–)	3.052	4017	2689	3	0
16	Archives of Diseases in Childhood (1926–)	3.041	16,291	22,375	53	7
17	Journal of Pediatric Gastroenterology and Nutrition (1982–)	2.937	12,405	10,860	12	0
18	Paediatric and Perinatal Epidemiology (1987–)	2.917	3398	2401	5	1
19	Pediatric Neurology (1985–)	2.890	5578	6348	4	4
20	Pediatric Critical Care Medicine (2000–)	2.854	6573	4892	1	1
21	Academic Pediatrics (2009–) Ambulatory Pediatrics (2001–2008)	2.810	2947	2770	56	0
22	Seminars in Pediatric Surgery (1992–)	2.807	1805	1349	0	0
23	Maternal and Child Nutrition (2005–)	2.789	3382	2210	6	3
24	Childhood Obesity (2010–) Obesity and weight management (2009) Obesity management (2005–2009)	2.756	1383	830	0	0
25	Pediatric Research (1967–) Annales paediatrici (1938–1966) Jahrbuch für Kinderheilkunde (1931–1937) Jahrbuch für Kinderheilkunde und physische Erziehung (1866–1930)	2.747	13,816	15,508	12	3
26	Neonatology (2007–) Biology of the neonate (1970–2006) Biologia Neonatorum (1959–1969)	2.742	2856	6072	1	0
27	Paediatric Respiratory Reviews (2000–)	2.716	1714	1798	2	1
28	Birth-Issues in Perinatal Care (1982–) Birth and the family journal (1973–1981)	2.705	2440	1905	2	1
29	Pediatric Nephrology (1987–)	2.676	9325	11,246	22	1
30	Frontiers in Pediatrics (2013–)	2.634	2922	3536	8	1

**Table 2:** Included papers by area, topic, and the primary or not primary outcome.

Area	Topic	Papers	Oral Health Primary Outcome	
			Yes	No
		*n* (%)	*n* (%)	*n* (%)
**Hard Tissues**	Caries prevention	85 (37.0)	74 (40.9)	11 (22.4)
	Caries treatment	33 (14.3)	20 (11,0)	13 (26.6)
	Caries epidemiology	65 (28.3)	51 (28.2)	14 (28.6)
	DDE	47 (20.4)	36 (19.9)	11 (22.4)
	**Total**	230 (100.0)	181 (100.0)	49 (100.0)
**Soft Tissues**	Oral aphthae	32 (51.6)	17 (42.5)	15 (68.2)
	Oral infections	6 (9.7)	6 (15.0)	0 (0.0)
	Gingivitis	10 (16.1)	9 (22.5)	1 (4.5)
	Other	14 (22.6)	8 (20.0)	6 (27.3)
	**Total**	62 (100.0)	40 (64.5)	22 (35.5)
**Orofacial Trauma**	Dental trauma	4 (22.2)	4 (26.7)	0 (0.0)
	Jaws trauma	2 (11.1)	2 (13.3)	0 (0.0)
	Combined trauma	5 (27.8)	2 (13.3)	3 (100.0)
	Suspected abuse	7 (38.9)	7 (46.7)	0 (0.0)
	**Total**	18 (100.0)	15 (100.0)	3 (100.0)
**Orofacial Development**	Syndromes	56 (72.7)	30 (61.2)	26 (92.9)
	Non-related to syndromes	21 (27.3)	19 (38.8)	2 (7.1)
	**Total**	77 (100.0)	49 (100.0)	28 (100.0)
**Public Health**	Health insurance	39 (33.3)	12 (16.0)	27 (64.3)
	Health services	64 (54.7)	52 (69.3)	12 (28.6)
	Pediatrician’s role	14 (12.0)	11 (14.7)	3 (7.1)
	**Total**	117 (100.0)	75 (100.0)	42 (100.0)

**Table 3:** Citations of the included papers by area, topic, and the primary or not primary outcome.

Area	Citations	Topic	Citations	Oral Health Primary Outcome	
				Yes	No
	*n* (*%*)		*n* (*%*)	*n* (*%*)	*n* (*%*)
	2762 (43.9)	Caries prevention	1121(17.8)	1883 (46.8)	879 (38.8)
**Hard Tissues**		Caries treatment	272 (4.3)		
*n* = 230		Caries epidemiology	690 (11.0)		
		DDE	679 (10.8)		
	736 (11.7)	Oral aphthae	489 (7.8)	437 (10.9)	299 (13.2)
**Soft Tissues**		Oral infections	15 (0.2)		
*n* = 62		Gingivitis	103 (1.6)		
		Other	129 (2.1)		
	82 (1.3)	Dental trauma	19 (0.3)	57 (1.4)	25 (1.1)
**Orofacial Trauma**		Jaws trauma	4 (<0.1)		
*n* = 18		Combined trauma	31 (0.5)		
		Suspected abuse	28 (0.5)		
**Orofacial Development**	679 (10.8)	Syndromes	593 (9.4)	300 (7.5)	379 (16.7)
*n* = 77		Non-related to syndromes	86 (1.4)		
	2028 (32.3)	Health insurance	888 (14.1)	1342 (33.4)	686 (30.2)
**Public Health**		Health services	883 (14.0)		
*n* = 117		Pediatrician’s role	257 (4.1)		
**Total**	6287 (100.0)		6287 (100.0)	4019 (100.0)	2268 (100.0)

## Data Availability

Data are available upon request. Please contact the corresponding author.

## Supplementary Materials

The following are available online at https://www.mdpi.com/article/10.3390/children8090720/s1, Table S1: PRISMA-ScR Checklist, Table S2: List of the excluded papers, Table S3: List of the included papers.
